# Nanoscale spectrum analyzer based on spin-wave interference

**DOI:** 10.1038/s41598-017-09485-7

**Published:** 2017-08-23

**Authors:** Ádám Papp, Wolfgang Porod, Árpád I. Csurgay, György Csaba

**Affiliations:** 1 Center for Nano Science and Technology University of Notre Dame (ND nano), Notre Dame, IN USA; 2 Pázmány Péter Catholic University, Faculty of Information Technology and Bionics, Budapest, Hungary

## Abstract

We present the design of a spin-wave-based microwave signal processing device. The microwave signal is first converted into spin-wave excitations, which propagate in a patterned magnetic thin-film. An interference pattern is formed in the film and its intensity distribution at appropriate read-out locations gives the spectral decomposition of the signal. We use analytic calculations and micromagnetic simulations to verify and to analyze the operation of the device. The results suggest that all performance figures of this magnetoelectric device at room temperature (speed, area, power consumption) may be significantly better than what is achievable in a purely electrical system. We envision that a new class of low-power, high-speed, special-purpose signal processors can be realized by spin-waves.

## Introduction

Magnetic excitations (spin waves) are one of the most promising ‘alternate state variables’ in electronics, due to their potentially very low energy, short wavelength, and high speed. A number of proposals and/or device demonstrations use spin waves for realizing Boolean logic gates^1, 2^ or non-Boolean computing primitives^2–9^. In this paper, we present a new class of devices and also a new application area for spin-waves. In particular, we show that they are very well-suited for high-frequency and extremely compact spectrum analyzer devices. Our device also exemplifies how ideas from non-Boolean, optical devices can be re-invented in the domain of spin-waves, which could be much more amenable to integration than light waves.

**Figure 1:**
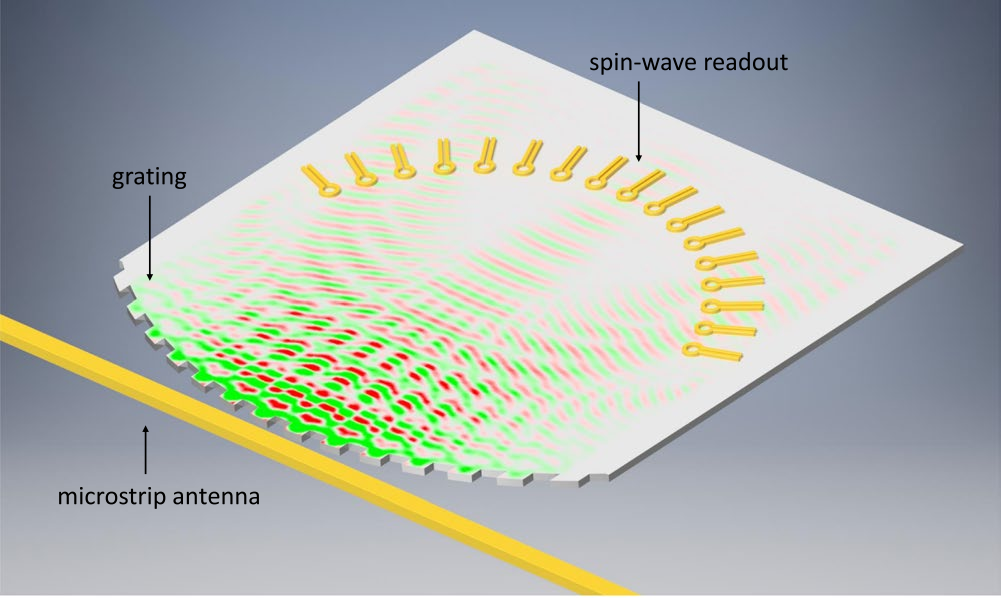
Schematic layout of the proposed spin-wave-based spectrum analyzer. The input signal is injected into a microstrip antenna, which generates spin waves on the patterned edge of an adjacent magnetic film. The interference pattern created by the spin waves is read out at specific locations using nanoscale loop antennas. The interference pattern is created in a way so that the frequency components of the input signal will become separated spatially.

In the studied device, the microwave signal is first converted into spin-wave excitations. The Oersted field of a simple waveguide can generate a coherent spin-wave wavefront in a magnetic thin-film. The signal processing takes place in the spin-wave domain, via linear interference, and the resulting interference pattern is picked up electrically at the output. The schematics of the spin-wave based processor is shown in Fig. 1. The wavelength of the studied spin-waves is two to six orders of magnitude shorter than the microwave wavelength at the same frequency, so switching to the spin-wave domain could enable very compact devices. The spin-wave wavefront carries energy that is about three orders magnitude less than the energy of the generating microwave signal. The heat dissipation of spin waves is very small in low-damping magnetic materials, indicating the potential of very low-energy processing. Spin-wave signals do not suffer from parasitic capacitive or inductive couplings, and the low magnetic damping of ferrite thin-films^10^ allows high signal integrity. The spectral analysis is done by spin-wave interference pattern formation. In effect, the interference pattern on the patterned film is functionally equivalent to a filter bank, made of high-*Q* factor *LC* components.

Microwave spectrum analysis is an essential capability in today’s telecommunications and electronic warfare systems, and also in many data processing applications. Due to the relatively long wavelength of microwave signals (centimeter range) and due to the fact that it is challenging to fabricate high-quality on-chip inductors and filters at higher frequencies, it remains challenging to fabricate fast, power efficient and high-resolution spectrum analyzers in a compact size. Digital spectrum analyzers can be made much more compact then passive ones, but they require high-speed analog-digital converters (ADCs) and such ADCs consume several watts of DC power. Frequency-domain processors, MEMS-based systems face similar challenges.

It must be noted that an on-chip, interference-based processor was recently proposed by Afshari *et al*.^11^–this work actually uses lumped *LC* elements to perform spectral analysis. However, due to the relatively large serial (parasitic) resistance of the inductors, this device is hard to scale to large sizes and consumes significant power. Here, we will argue that spin-waves may perform better in this aspect as well.

The use of magnetic materials in microwave signal processing and spectrum analyzers is not new. Magnetically tunable high quality oscillators can be made out of YIG (yttrium-iron-garnet) spheres with size under a millimeter^12^. Using YIG, filter banks and channelizers can be built, and exploiting the nonlinear property of the magnetic waves, frequency selective limiters have been demonstrated. Circulators and isolators typically use ferrites such as YIG. These devices, however, do not use magnetic excitations to actually carry information–rather, they use the magnetic materials as tunable permeabilities, exploiting the interaction of guided waves and magnetic excitations. A notable exception is the patent by Hanna *et al*.^13^, where beams of magnetic waves are deflected by a flat grating created by surface acoustic waves–we are not aware of any follow-up on this idea.

We believe that the only significant challenge in realizing these proposed type of magnetoelectric devices is the conversion of spin-wave signals back into the electric domain, which is required for most real-life applications. Due to the low energy and high frequency of the spin-waves, one needs fast, low-noise amplifiers for the magneto-electric conversion. Despite this challenge, spin-wave devices have the potential to combine ultra-low power and high-frequency operation, which is a virtually unmatched property among electronic/nanoelectronic devices.

### Principles for wave-based spectral analysis

At the heart of the proposed device is a concave grating, that serves dual purpose: (1) it creates a diffraction pattern and (2) it focuses the waves. Concave gratings are traditionally used in optical and x-ray spectroscopy, typically in Rowland circle spectrographs^14, 15^. Concave gratings offer several advantages over conventional flat gratings by eliminating the need for lenses in the system–lenses are often difficult to realize outside the optical domain.

**Figure 2:**
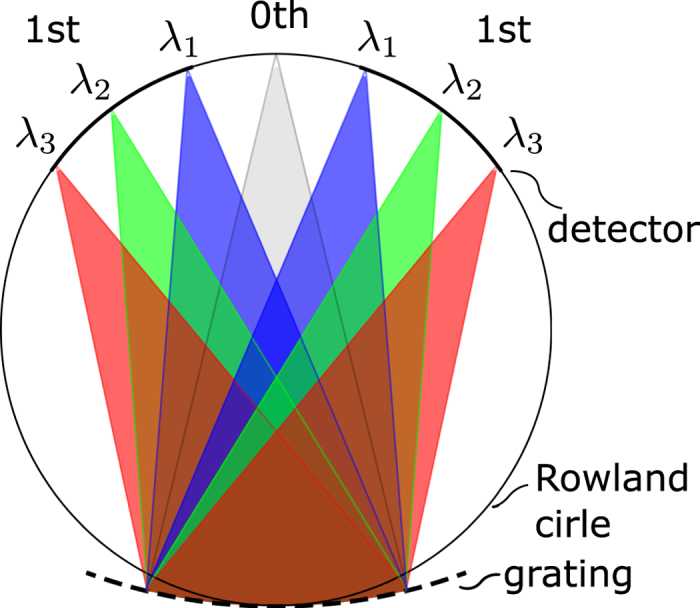
Schematic layout of a Rowland spectrograph. A multispectral time-domain signal excites waves at the grating, and the wavefronts interfere in such a way that the spectral decomposition of the signal appears as interference maxima on the Rowland circle.

A schematic sketch of a generic Rowland circle spectrograph is shown in Fig. 2. At the bottom of the device is the curved grating. This diffracts waves with different wavelengths along different directions, and–due to its concave shape–also focuses them to different points on the Rowland circle, as indicated in Fig. 2. Since the wavelength depends on the excitation frequency, different frequency constituents of a time-domain signal will launch waves with different wavelengths. So if a signal with multiple time-domain spectral components is launched from the grating, then the intensity distribution along the Rowland circle will give the spectral decomposition of that signal.

**Figure 3:**
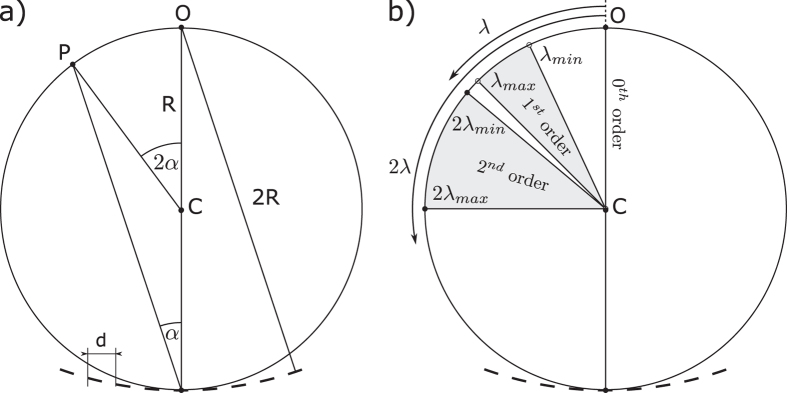
(**a**) Sketch for calculating the resolution of the Rowland circle arrangement. (**b**) Position of the diffraction orders around the Rowland circle. *λ*_*min*_ and *λ*_*max*_ are the minimal and maximal wavelength components in the input signal. In order to avoid band overlap, *λ*_*max*_ < 2*λ*_*min*_ must be fulfilled.

The spectral resolution of the device depends on the spatial separation of the spectral components on the Rowland circle. The deflection angle *α* (as seen in Fig. 3a) of a wave with wavelength *λ* on a grating with grating constant *d* is given by the grating equation^14^:

1 $$\sin \,\alpha =\frac{n\lambda }{d}$$

where *n* is the diffraction order. This formula is valid for flat gratings and a good approximation for gratings with small curvature, which is the case we consider here. In case of a concave grating, the deflection angle *α* is the same at every point on the grating and the waves with same wavelength are focused to a single point *P* on the Rowland circle (see Fig. 3a). It is easy to see that ∠*PCO* = 2*α* so the length of the arc is:

2 $$\mathop{PO}\limits^{\frown {}}=2R\,\arcsin \frac{n\lambda }{d}.$$

Thus, for small angles, the position of the focal point *P* on the Rowland circle depends approximately linearly on the wavelength *λ*. The resolution of the system can be engineered by choosing the appropriate Rowland circle radius, *R*. Note, that any wave with a wavelength *λ* ≥ *d* will have only the trivial zero order component.

Figure 3(b) illustrates the location of the higher-order peaks. In the design of the spectrum analyzer, care must be taken to avoid overlap between different diffraction orders and this limits the operation of the device to a frequency band between the corresponding *λ*_*min*_ and *λ*_*min*_ wavelengths. For correct operation, the input signal can not contain high frequency components with spin-wave wavelength less than λ_min_, thus the input signal must be pre-processed (or post-processed) by a low-pass filter. The lowest frequency that can be distinguished without second order overlap corresponds to *λ*_*max*_ = 2*λ*_*min*_.

The calculations presented so far use only the wavelength and assume generic linear wave properties. One needs to know the dispersion relation (wavelength-frequency relation) in order to determine the spectrometer resolution in terms of frequency. For electromagnetic waves, there is usually a linear relation between wavelength and frequency, while spin waves have a more complex, nonlinear dispersion relation.

### Spin-wave propagation in magnetic thin films

Magnetic materials can be thought of as an assembly of interacting, elementary magnetic moments (spins). In a classical picture, a space and time-dependent, continuous magnetization distribution **M**(**r**,**t**) characterizes a ferromagnetic (or ferrimagnetic) material^16^. The spins are coupled to each other via magnetostatic and exchange interaction. Disturbances in the magnetization distribution propagate in a wave-like manner and are referred to as spin waves or magnons. For small amplitudes (few degree deflection of the **M** vector), spin waves behave as linear waves to a good approximation.

There are also major differences between spin waves and electromagnetic waves. The spin-wave dispersion relation strongly depends on material parameters and externally applied magnetic fields and may be ‘engineered’ by an appropriate choice of these parameters. Spin-wave wavelengths as small as a few-ten nanometers may correspond to microwave frequencies (5–100 GHz). Spin-wave propagation is often anisotropic and the wavelength depends on the relative orientation of **M** magnetization and the **k** wavevector. Spin waves are strongly damped in most ferromagnetic metals, but they may propagate large distances (thousand times wavelength) in ferrites (such as yttrium iron garnet, YIG)^17^.

Spin-wave dynamics can be modeled by standard micromagnetic theory and a number of established software packages are available for this task^18^. Most of these simulators solve the Landau-Lifshitz-(Gilbert) equations (LLG equations) in the time-domain. For certain spin-wave propagation modes and certain parameters one can often linearize the LLG equations and/or find analytical solutions for the dispersion relation^19^.

As an example, we consider dispersion relation for spin waves in a film that is magnetized along the $\widehat{Z}$ direction^16^:

3 $$\omega =\sqrt{({\omega }_{0}+{\omega }_{M}{\lambda }_{ex}{k}^{2})({\omega }_{0}+{\omega }_{M}({\lambda }_{ex}{k}^{2}+si{n}^{2}\theta ))}$$

where *ω*_0_ = *γμ*_0_ *H*_0_, *ω*_*M*_ = *γμ*_0_ *M*_*S*_, ${\lambda }_{ex}=\frac{2{A}_{exch}}{{\mu }_{0}{M}_{S}^{2}}$, *γ* is the gyromagnetic ratio, *μ*_0_ is the vacuum permeability, *M*_S_ is the saturation magnetization, *H*_0_ is the total internal field, *A*_*exch*_ is the exchange coefficient, *k* is the wavenumber and *θ* is the angle between the propagation direction and $\hat{{\bf{z}}}$.

One may distinguish between two fundamentally different propagation modes of spin waves. For waves with large *k* (short wavelength), the term *λ*_*ex*_ *k* ^2^ in Eq. 3 is much smaller than one and is negligible compared to the other terms. Waves in this parameter regime are called exchange waves because the dominant interaction mechanism between oscillating spins is the exchange interaction. On the other hand, if *λ*_*ex*_ *k* ^2^ **≫** 1 (long wavelength), then the dipole interactions dominate, and such waves are called dipole spin waves or magnetostatic waves. Exchange-waves have several orders of magnitude shorter wavelengths compared to electromagnetic (EM) waves at the same frequency, but even magnetostatic wave wavelengths are at least two orders of magnitude shorter than EM wave wavelengths. This makes spin waves attractive to use in compact (on-chip) applications.

The operation of the device proposed here relies only on the interference of linear waves and the device may be designed to work either in the exchange-dominated or the dipole-dominated spin-wave regime. Both modes of operation have benefits and drawbacks. Exchange waves allow much more compact devices, but make device fabrication and detection of spin waves more challenging. The benefit of short wavelengths is that if the size of the entire device is smaller than the electromagnetic wavelength, then one does not need to worry about possible phase delays occurring in the driving microwave circuitry. It is worthwhile to mention that passive microwave components (filters, spectrum analyzers, surface acoustic wave devices, etc.) are rather large by the standards of microelectronics^20^. So, even if one assumes relatively long spin wave wavelengths (on the order of several micrometers or more), this will still result in a device that is compact compared to most electrical implementations. The dispersion relation of Eq. 3 depends on the angle (*θ*) between the direction of wave propagation and the magnetization. This dependence results in anisotropic wave propagation for in-plane magnetized films, but not in out-of-plane films, since in that case the out-of-plane (normal) vector is perpendicular to every wavevectors in plane. In the following, we assume out-of-plane magnetized films in order to avoid complications that arise from anisotropic propagation.

**Figure 4:**
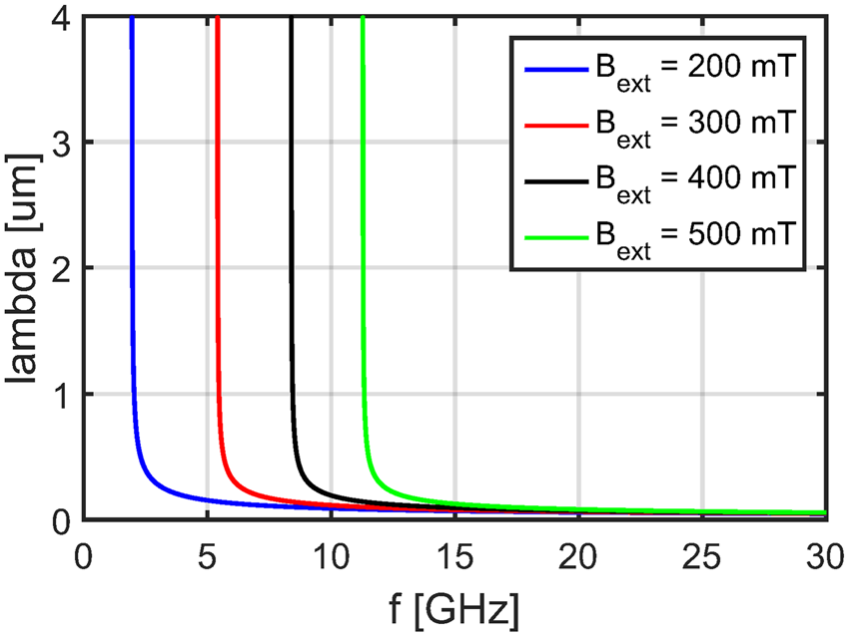
Calculated dispersion relation of spin waves in an out-of-plane magnetized YIG thin film assuming various external fields.

In the previous section, we showed that in the Rowland configuration there is an approximately linear mapping between wavelength and the location of the corresponding focus point on the Rowland circle (Eq. 2). In order to determine the mapping of frequencies, Fig. 4 shows calculated dispersion curves in a thin YIG film at various ***B***_*ext*_ external fields. It is possible to set *B*_*ext*_ such that the frequency band of interest falls on a higher or lower slope of the dispersion curve. By doing so, one may achieve a narrow-band spectrometer with high frequency resolution, or wide-band spectrometer with lower frequency resolution while other design parameters remain unchanged. A given geometry (i.e. given *d*, *R*) together with *λ* wavelength will result in given a wavelength resolution for a certain device. The corresponding frequency resolution depends on the dispersion relation - and one can use the *B*_*ext*_ field as a design parameter. The spectral range is determined by the frequency resolution and the number of output points, likely the latter one being the more important practical limitation.

As a concrete numerical example, we assume *d* = 4 *μ*m and *R* = 1 mm. The first order peak of a *λ*_1_ = 1 *μ*m spin wave will be focused at $\widehat{{P}_{1}O}=505.4$ *μ*m. The wavelength of a wave focused in the next output point at $\widehat{{P}_{2}O}=509.4$ *μ*m will have a wavelength *λ*_2_ = 1.008 *μ*m. If a bias field *B*_*ext*_ = 300 mT were applied, these wavelengths correspond to *f*_1_ = 5.465 GHz and *f*_2_ = 5.464 GHz, i.e. a frequency resolution Δ*f* = 1 MHz corresponds to a *l* = 4 *μ*m distance on the Rowland circle.

If the spectrometer is designed to operate in the spin-wave wavelength regime between *λ*_min_ = 1 *μ*m and *λ*_max_ = 2 *μ*m, then the first readout antenna have to be placed to *α* = 14.48°, or $\widehat{{P}_{min}O}=505.4$ *μ*m. According to Eq. 1, to avoid overlap of the first and second order peaks, the highest deflection angle where a detector can be placed is $arcsin\frac{2{\lambda }_{min}}{d}={30}^{\circ }$, and this corresponds to $\widehat{{P}_{max}O}=1047.2\,\mu$m. Thus, the number of output points will be $\frac{\widehat{{P}_{min}{P}_{max}}}{l}=135$. The minimum wavelength will give the maximum frequency *f*_*max*_ = 5.465 GHz, and the maximum wavelength will correspond to the lowest value in the frequency band, *f*_*min*_ = 5.418 GHz, i.e. in our example, the spectral range will be 47 MHz, with the Δ*f* frequency resolution changing from Δ*f* = 1 MHz to Δ*f* = 0.2 MHz along the arc. By designing the device to work on a lower slope of the dispersion curve, the spectral range can be increased to the gigahertz range, at the expense of frequency resolution. One also has to use shorter wavelengths in a wider-band device. The most important practical limitation of the device is probably the number of output points, which can only be increased by increasing the device size (*R*) and/or decreasing the *l* distance between readout points.

Magnetic damping will limit the spin-wave propagation length and diminish signal integrity. YIG has one of the lowest damping among the known magnetic materials, with damping coefficient as low as *α* = 8.58 × 10^−5^ ^10^ for thin films and decay lengths of up to thousand wavelengths^17^ - albeit one has to consider that this value may significantly decrease due to patterning^21^. Thus the device in the above numerical example is realizable with sufficiently strong spin-wave signal reaching the Rowland circle.

### Generation of spin waves on a grating

In a conventional Rowland-circle spectrograph, an optical or X-ray beam is directed to the grating, which acts as a secondary source of waves. In the case of spin waves, the grating can be made the primary source of the spin waves.

**Figure 5:**
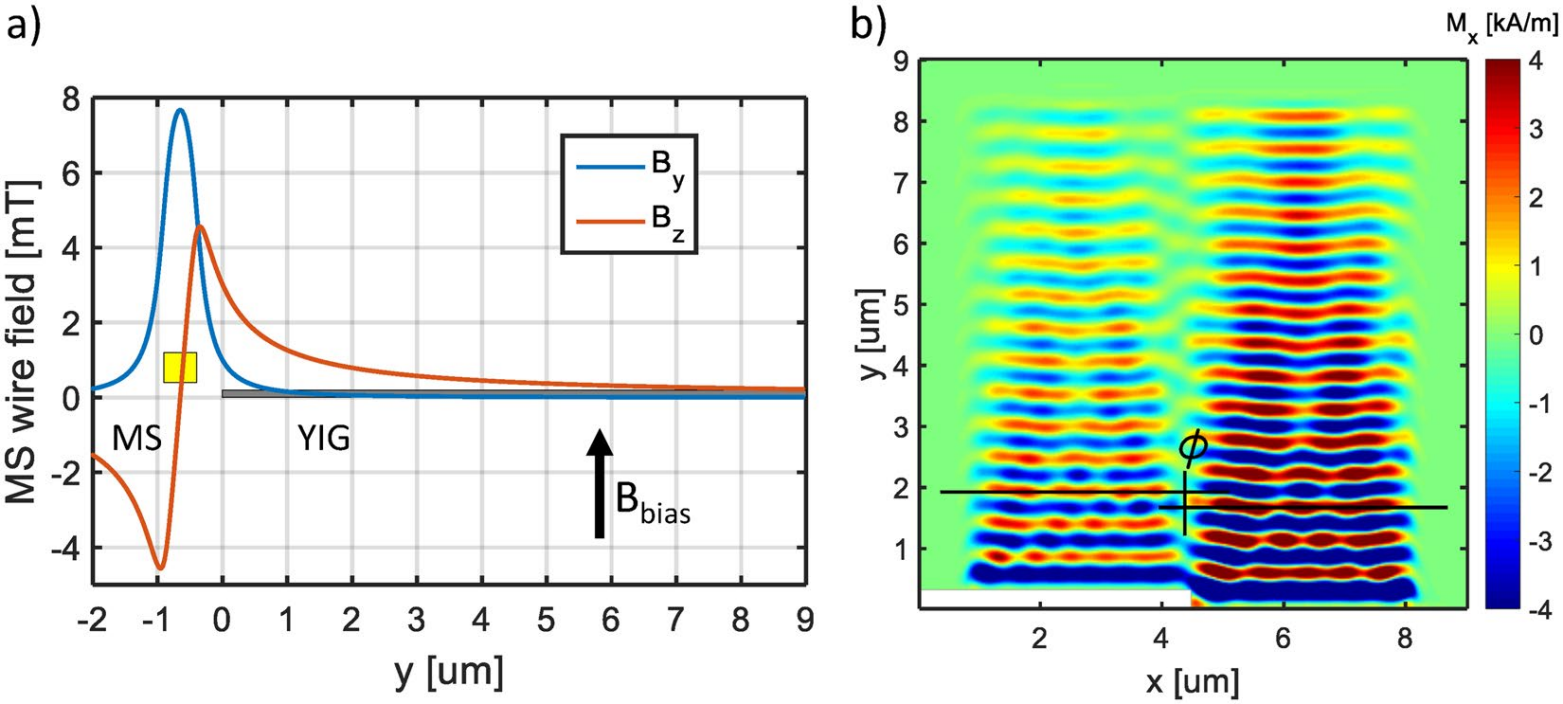
Spin-wave generation on the edge of a YIG film by a microstrip line (MS). (**a**) Magnetic field of MS and schematics of arrangement. (**b**) Time snapshot of the magnetization of YIG showing spin waves generated on the edge close to the MS (bottom). By patterning the film edge phase shifts may be introduced to the coherent wavefront.

The edge of a magnetic film, with a microstrip (MS) line running next to it, can act as the source of spin waves. The geometry is illustrated in Fig. 5a. The Oersted field of the current that runs through the microstrip line generates spin waves at the edge of the film.

The use of coplanar or microstrip waveguides for the generation of spin waves is well-established^22^. The magnetic field of such waveguides is not well-localized. Waveguides are rather inefficient in creating spin waves with wavelengths that are smaller (or comparable) to their width. However, at the edge of a magnetic film, the demagnetization field changes abruptly. It is the net magnetic field (which is the superposition of demagnetization field, waveguide-generated Oersted field and other effective field components) that is responsible for the generation of the spin waves. We found that the abruptly-changing field at the edge of the waveguide is a significantly more efficient coherent source of spin waves than the waveguide alone.

Generating spin waves at and by the boundary of the magnetic film has another significant benefit: it enables precise phase-shifting of the waves by patterning the edge. A diffraction grating for micrometer-wavelength spin waves is able to introduce an initial phase shift at the spin-wave generation.

An example of the phase-shifting edge is shown in Fig. 5b. The *h* = 270 nm step was designed to introduce an initial phase difference of *ϕ* = *π*. The width of the waveguide in this example is *w* = 500 nm and its calculated magnetic field is depicted in Fig. 5a. The magnetic field of the MS line was calculated numerically by integrating the field components generated by small sections of the MS wire using Amperes law. In this simplified simulation model, we assumed uniform current distribution and neglected the effect of the ground plane and the dielectric. The calculated field is concentrated around the MS wire, where the magnetic film edge is located. Consequently, the magnetic film edge experiences almost exactly the same field at the two sections of the step, but in one side the source is shifted by the *h* step size resulting in a phase shift.

A periodic structure built from the steps of Fig. 5b, next to the waveguide, can simultaneously act as the spin-wave source and diffraction grating in a spin-wave-based Rowland circle.

### Micromagnetic simulation of the spectrum analyzer

We used micromagnetic simulations (OOMMF^18^) to verify and demonstrate the design above. OOMMF solves the Landau-Lifshitz-Gilbert equations in the time domain. This approach is based on the fundamental equations of micromagnetics and avoids most approximations–but is computationally intensive. For two-dimensional structures typically a few micrometer by few micrometer size structure is reasonable to simulate.

We simulated a 10 nm thick YIG film with saturation magnetization *M*_s_ = 1.4 × 10^5^ A/m, exchange constant ${A}_{exch}=3.65\times {10}^{-12}$ J/m and *α* = 0.001 damping constant. This damping constant is much higher than what is achievable in state of art YIG thin films, but we assumed a pessimistic value, and the larger damping constant also made the numerical calculation more stable.

We chose a lateral cell size of 15 nm–while this is a relatively coarse discretization, it allows the simulation of a 15 × 15 *μ*m area on an average workstation and within a few days of simulation time. In order to verify the accuracy of this cell size, we performed simulations on similar structures using much finer discretization (all the way down to 5 nm) and on much coarser grids. The simulations gave nearly identical results, as long as the cell size was small compared to the spin wave wavelength studied. The out-of-plane external bias field was set to *B*_*bias*_ = 520 mT, which according to the dispersion relation corresponds to a wavelength of *λ* ≈ 525 nm at a frequency *f* = 10 GHz.

The geometry of the magnetic film follows the design described above: at the bottom of the structure there is the curved grating, serving also as the source of spin waves. This edge of the YIG film was patterned in a cogged shape on an arc with a radius of 2*R* = 12 *μ*m, where *R* is the Rowland circle radius. The grating constant was set to be equal to double the wavelength *d* = 2*λ* at 10 GHz and the height of the cogs is *h* = *λ*2, which equals a phase shift of *π*.

The input signal, which is the magnetic field of the waveguide, appears in the simulation as a time varying external magnetic field. In the present example, this field is a superposition of two sinusoidally varying magnetic fields at *f*_1_ = 10 GHz and *f*_2_ = 10.25 GHz. The field distribution of the waveguide was calculated numerically in an independent simulation as described previously, assuming a microstrip line with width *w*_*ms*_ = 500 nm, thickness *t*_*ms*_ = 200 nm and dielectric thickness *t*_*diel*_ = 100 nm. The microstrip was placed 400 nm from the YIG film. The microwave current amplitude in the line for both frequency components was 0.5 mA. We calculated the the current distribution and the waveguide magnetic field using HFSS a full-wave electromagnetic simulator. We ignored the influence of the magnetized film on the waveguide.

On the other three edges of the YIG film absorbing boundary conditions were realized by a linearly increasing damping coefficient in a 1500 nm region up to a maximum damping coefficient *α* = 0.5. This boundary condition eliminates most reflections from these edges, effectively simulating an infinitely extended film in these directions.

The simulation was performed in two steps: in an initial simulation only the bias magnetic field was applied, without the oscillating field component and the damping constant was set to *α* = 0.5 everywhere. With the artificially high damping constant, the simulation quickly converges to a steady-state **M**(***r***) magnetization distribution. In the next step, the damping constant is set to its real, low value and the oscillating field of the waveguide is applied. The spin waves appear as small (few-percent) perturbations on top of the previously calculated magnetization distribution.

**Figure 6:**
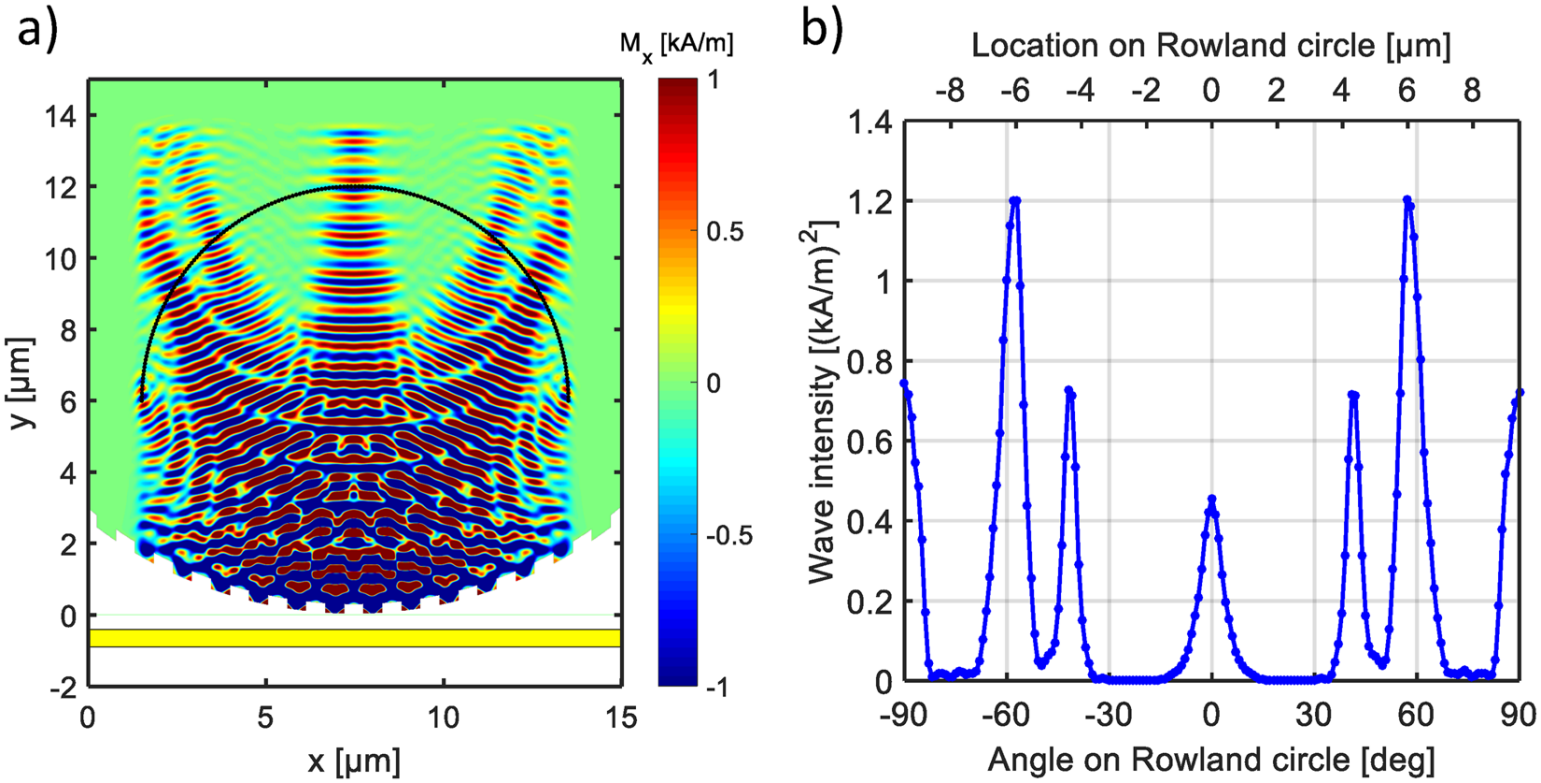
Micromagnetic simulation of the spin-wave-based Rowland circle spectrometer. (**a**) The colormap shows a magnetization snapshot of a YIG film, the peaks on the Rowland circle correspond to frequencies *f*_1_ = 10 GHz and *f*_2_ = 10.25 GHz. The yellow stripe at the bottom is a sketch of the microstrip that is used as a source. (**b**) Spin-wave amplitude along the Rowland circle indicated by black arc in (**a**).

Figure 6 shows a snapshot of the magnetization distribution from the simulation. It is a contour plot of the *M*_*x*_ magnetization component (*M*_*y*_ would look similar). An interference pattern is formed, as expected from the theory of the Rowland spectrograph. The results confirm that in the chosen parameter regime, the spin waves behave exactly as expected from the simple picture based on linear waves.

Figure 6b shows the amplitude of waves in the simulation on the Rowland circle. The finite width of the peaks is caused by diffraction, which should set the width of peaks to be approximately equal to the wavelength, and this is exactly what is seen in the output intensity distribution of Fig. 6. In the simulated device, this allows to resolve only a few (approximately four) peaks–but this is due to the very small simulation domain.

The amplitude of the two frequency components in the microwave signal were set to be equal, but one can see in Fig. 6 two different spin-wave amplitudes corresponding to these frequencies. This is due to the geometry of the grating we used. On this grating, all wave components experience the same periodic shifting in distance, but this distance shift corresponds to different phase shifts depending on the wavelength. Thus the grating is not equally effective at every wavelengths, which results in different amplitudes. Spin-wave-generation efficiency of the waveguide also becomes smaller at shorter wavelengths, and the damping can also play a role in large device sizes. In the example shown here, a 250 MHz frequency separation in the input signal translates to a factor of approximately 1.5 change in the spin-wave wavelength. This, in turn, results in less than a factor of two difference in the intensity of the peaks at read-out. Overall, the net change in the output intensity always remained below a factor of three in our simulations. Still, these effects have to be taken into account by proper scaling (calibration) after the read-out.

For most practical purposes, one will have to use a device that is few hundred to few thousand times the size of the chosen spin wave wavelength. Direct micromagnetic simulation of structures with such size is not possible, but we expect that scaling up the device does not bring in unexpected effects and the conclusions we have drawn from the above simulations remain valid. However, one has to pay particular attention to the design of the microwave interfaces, such as the input waveguide. Microwave phase shifts may occur (1) along the waveguide; and (2) due to the varying distance between the curved edge of the film and the straight waveguide. The (1) effect caused by the waveguide length is a few times larger than effect (2). This is due to the slower microwave phase velocity along the waveguide and to the small arc length of the grating - assuming a grating arc length of 60°, the change of distance between a straight waveguide and the curved film edge can be calculated as $2R\mathrm{(1}-\cos \,{\mathrm{(30}}^{\circ }))$, which in case of *R* = 1 mm will be 268 *μ*m. Overall, microwave phase shifts on the order of 100 *μ*m to millimeter scale may result in spin-wave phase shifts up to 10–100 nm, which may be non-negligible (i.e. comparable to the spin-wave wavelength) and could possibly blur the interference pattern. The phase shift caused by (2) may straightforwardly be alleviated by a curved waveguide, which keeps a constant distance from the film edge.

The curved grating is central to the function of the device and this is the component that requires high-quality, nanoscale fabrication resolution. If the boundary of the grating is different from the ideal, rectangular shape (e.g. all edges are rounded and not sharp), but the structure is periodic to a good approximation, then these variations will only change the power distribution between different-order peaks. Non-periodic changes in the shape will degrade the spectral resolution. Since the wavelength range is a design parameter, one may choose to use longer-wavelength (dipole-dominated) waves if high-resolution patterning of the magnetic film turns out to be difficult of overly expensive. YIG patterning in notoriously challenging, but is demonstrated for hundred-nanometer size scale features^21^. One could also imagine other types of grating structures for generating and manipulating the spin waves^23^.

For the device to work as a Rowland spectrograph, spin waves should display linear interference pattern–and this requires proper setting of the excitation amplitude. In order to estimate the excitation range, where (approximately) linear waves are generated, we analyzed spin wave propagation in various one-dimensional structures using numerical simulations. We choose different excitation amplitudes in a similar antenna geometry to the one described here. The spectral decomposition (Fourier transform) of the time-dependent magnetization was studied at certain points in the simulated structure. We found that the harmonics (i.e. spectral components different from the excitation frequencies) remained negligibly small all the way up to excitation currents four times four times larger than the ones used in the simulations. By negligibly small we mean that the amplitudes remained below one percent of the main peak. In the presented simulations, the maximum precession angle (at the point of spin wave generation) is around five degrees and with these parameters waves remain safely in the linear regime.

All simulations were done at *T* = 0 K, i.e. they do not take into account thermal agitation of the magnetic moments. Thermal fluctuations appear as a wideband noise on top of the spin-wave signal and they superpose to other noise sources, such as the Johnson-Nyquist noise in the electrical components. Preliminary calculations show that the magnetic noise will be negligibly small compared to Johnson-Nyquist noise in the magnetoelectrical interfaces^24^.

### Magneto-electrical interfaces

A practical signal processing device has electrical inputs and outputs–even if signal processing itself is done outside the electrical domain. Conversion of electrical signals into spin-wave signals is the easier part as a relatively simple waveguide geometry may be used. But energetically, this is an inefficient process. In the literature of YIG-based devices, one typically finds that magnetostatic waves appear as a few-hundred ohms per centimeter load to waveguides^25^–this means that for the at most millimeter-sized device proposed here, the he magnetic film represents a rather small load to the waveguide. Most microwave energy is dissipated at the load terminating the waveguide and only a few percent of the microwave energy is converted into spin waves. Still, only a few milliwatts of microwave power is required for wavefront generation, which is very small compared to the often several-watts consumption of microwave devices. There are potentially more power efficient and more practical ways for spin wave generation: magneto-elastic effect is a promising, demonstrated technique that may substitute the waveguide-based generation^26^. Spin-orbit torque (Spin Hall Effect)^27^, possibly used with a patterned waveguide could be especially practical for short-wavelength spin wave generation. Spin-orbit torque, with sufficiently anisotropic materials could also make the external field biasing unnecessary^27^. Spin-torque is an established method for short-wavelength spin wave generation^28^, albeit less trivial to use in magnetic insulators such as YIG^29^. A more significant challenge, however, lies in converting the very low-energy spin-wave signals back to the electrical domain. Read-out of spin waves is possible by micron-scale antennas or using the inverse spin Hall effect (iSHE)^30^. In either case, the output signal will be in the few-ten microvolt range^24^. For the antenna-based pickup, it will be an AC voltage, while iSHE results in DC output.

For antenna-based pickup of spin waves, we presented a case study in^24^. The specifications and engineering challenges for the pickup circuitry are very similar to what one faces in the first stages of a microwave/radio-frequency front end. For few-micrometer sized antennas, one expects microvolt signal levels. Receiving and amplifying microvolt signal levels at these frequencies requires significant circuitry - both amplifiers and filters that restrict the bandwidth in order to limit resistive noise. Similar pickup circuits were already realized for different purposes^31^ and the possibility of using CMOS up to 100 GHz^24^ certainly boosts the practicality of such a solution.

Inverse Spin-Hall effect-based readout could be more practical as the resulting DC voltage can be detected with slower amplifiers and no filtering and mixing is required^30^. Most likely, however, one needs circuit techniques to eliminate DC drifts, and if high output date rate is required, resistive noise will limit the dynamic range of the read-out. Regardless of the chosen method, the output signal is proportional to the area where spin wave intensity has to be detected, and the magnetoelectric conversion will be more challenging for the short wavelength, exchange dominated regime.

Most proposed spin-wave-based logic or computing devices require magnetoelectric conversions between gate-level building blocks^2^, which results in a large input/output overhead - outputs are the Achilles heel of all spin-wave based devices. The device we propose here performs a rather complex signal processing function with few inputs and outputs, amortizing the large cost of magnetoelectric interfaces.

### Conclusions and outlook

New nanoscale computing devices often target power-efficient switches that could possibly replace CMOS devices for future circuits and keep Moore’s law going^2^. However, it is a tall order to beat CMOS devices in all figures of merit. Sub-threshold CMOS devices could be extremely power efficient albeit slow–but one can trade power for high-speed operation, if needed. In this paper, we argued that high-speed and low-power special-purpose processing may be an application area where spin waves may significantly outperform electrical-circuit-based solutions. The proposed signal-processing also exemplifies a non-Boolean computing primitive–a special-purpose computing task, which is not computationally universal, but can be immensely useful in many applications. One may envision similar optically inspired spin-wave devices, such as lenses and mirrors to perform Fourier transformation and filtering^9^, or holographic pattern matching.

Microwave signal processing, up to possibly several hundred gigahertz frequencies could be a real market niche for spin-wave-based processors^32^. Most spin (wave) based device proposals target computing and logic applications, competing with proven CMOS-based approaches. Microwave structures may turn out to be a natural application area for spin waves and an area where no competing transistor-based solutions exist.
